# Supplementary figures and images for: Construction, characterization, and immunization of nanoparticles that display a diverse array of influenza HA trimers
Source: PLoS One. 2021 Mar 4;16(3):e0247963. doi: 10.1371/journal.pone.0247963 (PMC7932532; doi:10.1371/journal.pone.0247963)

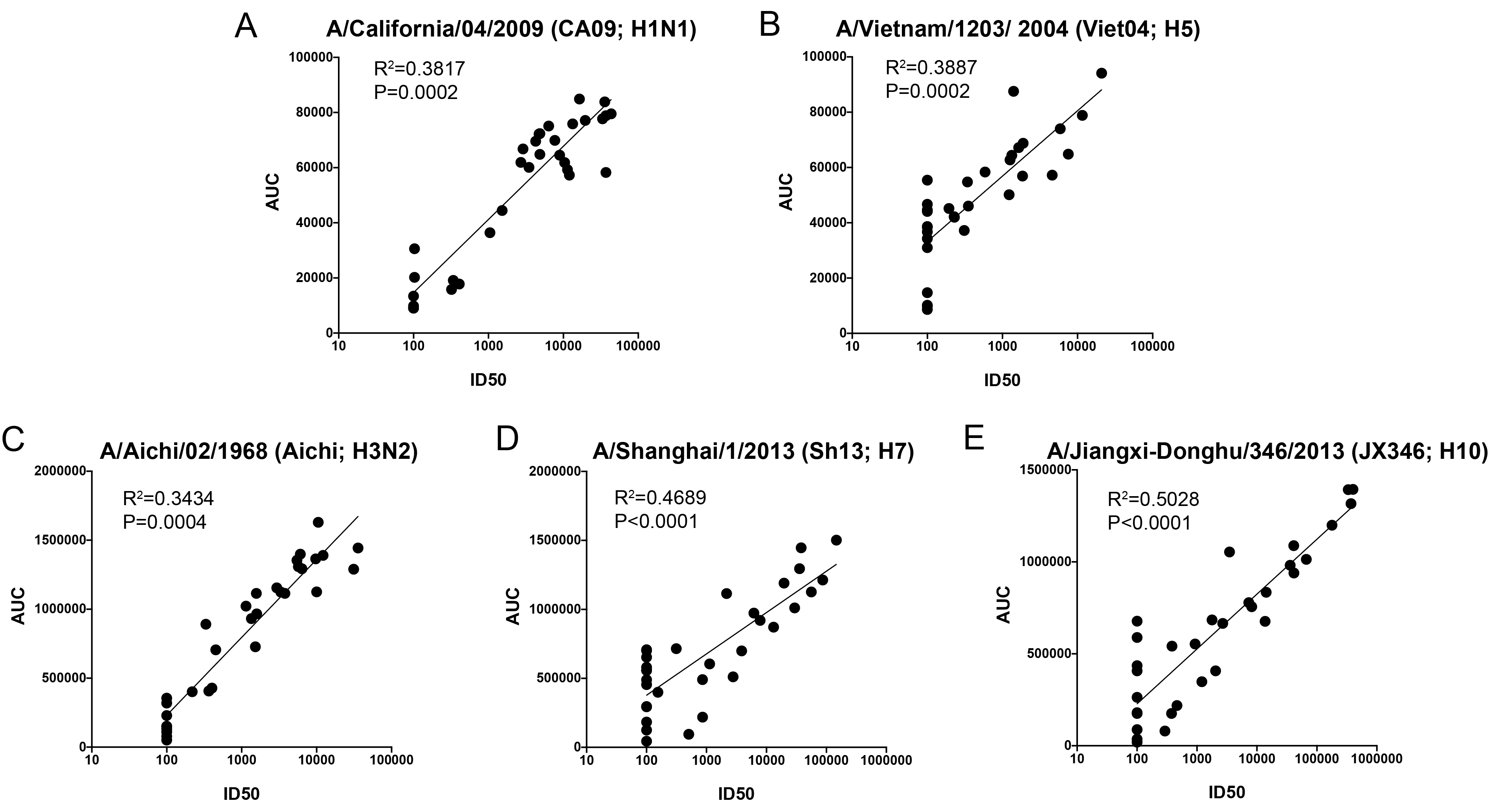

Supplement: S1 Fig — Pearson correlation of Day45 serum ELISA AUC titers and viral neutralization titers for A. CA09 H1N1 B. Viet04 H5 C. Aichi H3N2 D. Sh13 H7 E. JX346 H10. (PNG) [file pone.0247963.s001.png]

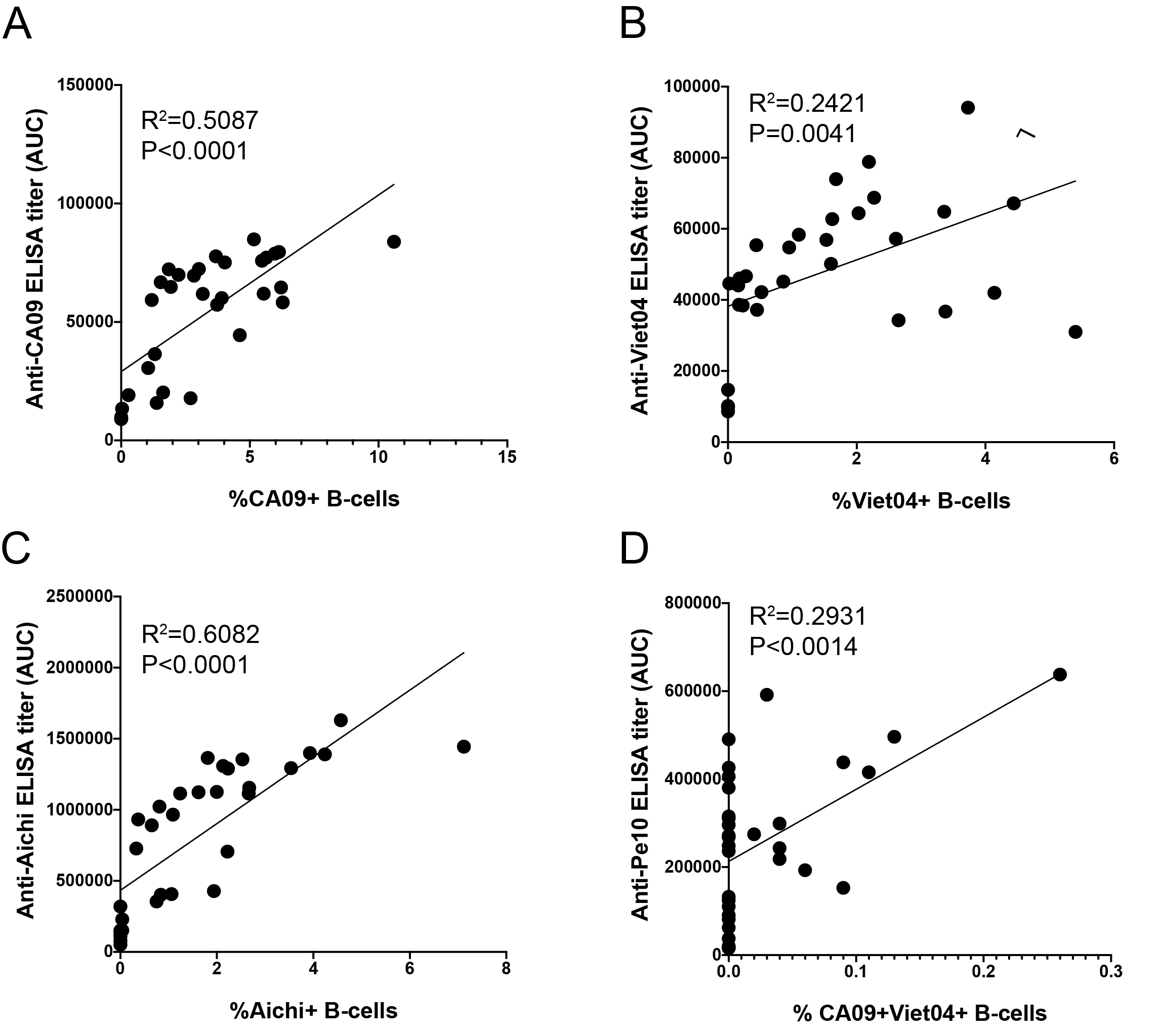

Supplement: S2 Fig — A. Pearson correlation of Day45 CA09+ B-cell population to serum anti-CA09 ELISA AUC titers. B. Pearson correlation of Day45 Viet04+ B-cell population to serum anti-Viet04 ELISA AUC titers. C. Pearson correlation of Day45 Aichi+ B-cell population to serum anti-Aichi ELISA AUC titers. B. Pearson correlation of Day45 CA09+Viet04+ B-cell population to serum anti-Pe10 ELISA AUC titers. (PNG) [file pone.0247963.s002.png]

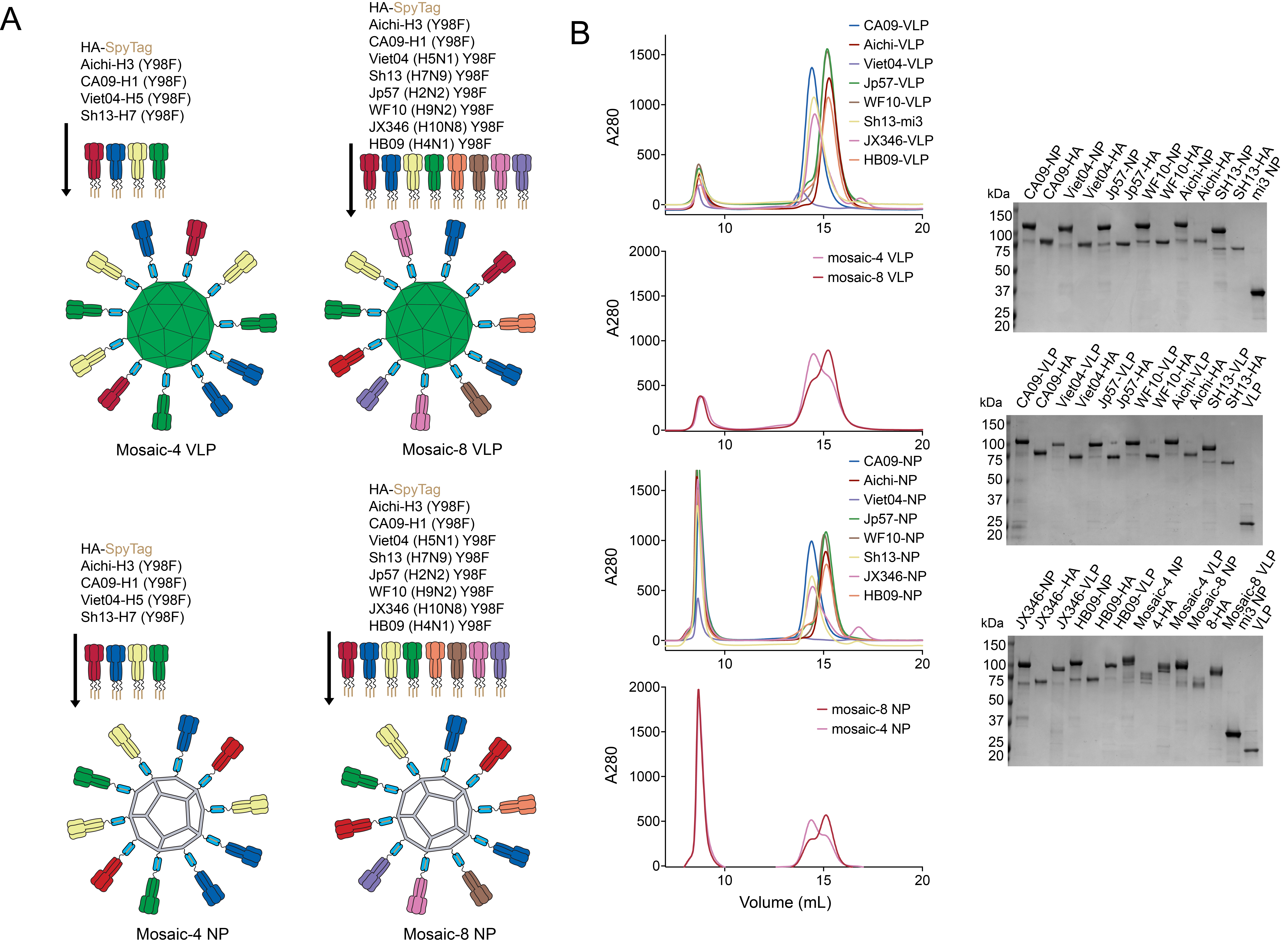

Supplement: S3 Fig — A. SpyCatcher-AP205-VLP and SpyCatcher-mi3 conjugations with SpyTagged-HA trimers. B. Purification of homotypic and mosaic SpyCatcher-VLPs and SpyCatcher-mi3s. Left: SEC separation of conjugated NPs from free HA trimers. Right: Reducing SDS-PAGE analysis of NPs and purified HAs. (TIFF) [file pone.0247963.s003.tiff]

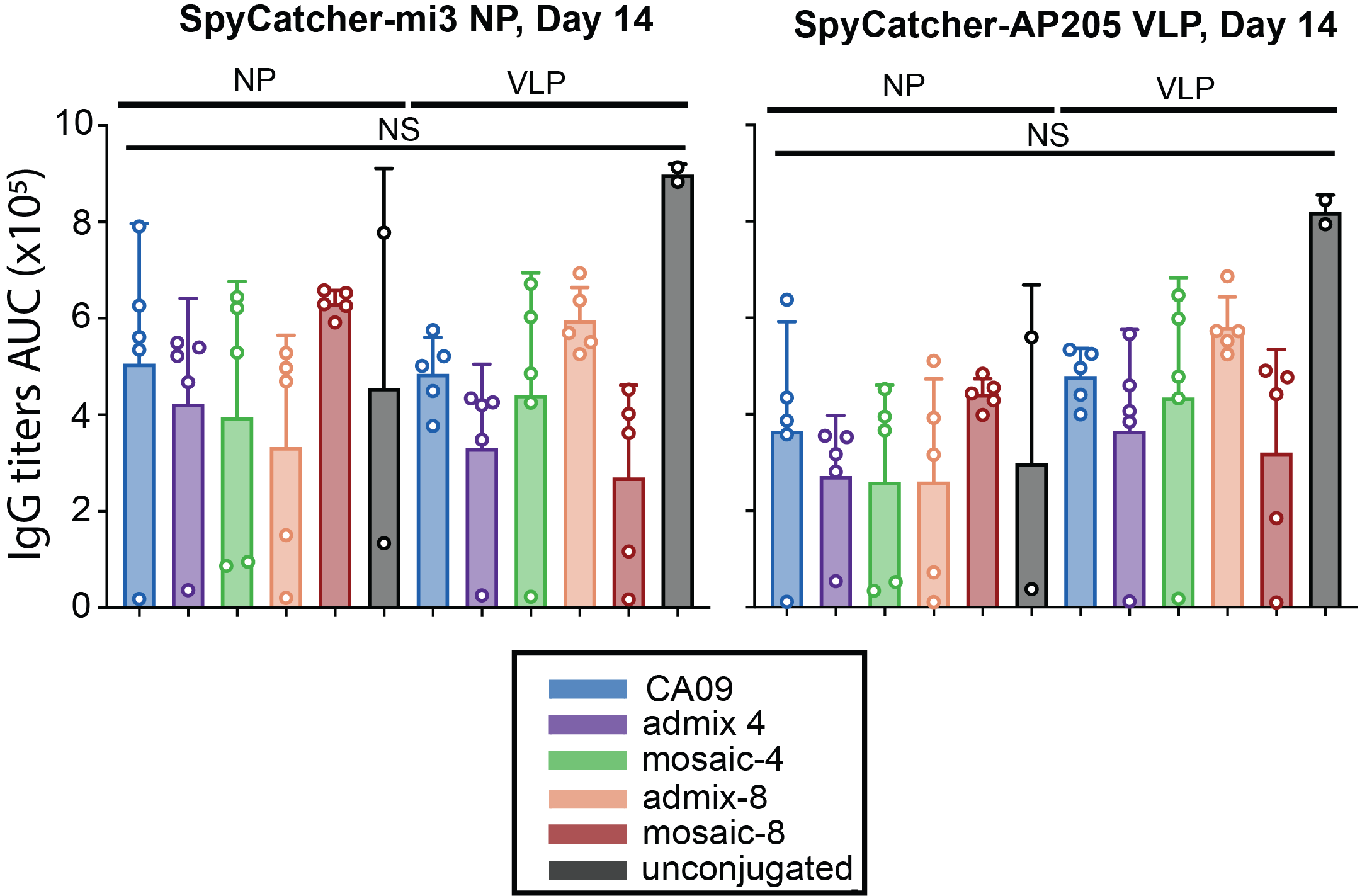

Supplement: S4 Fig — Serum antibody response to HA shown by ELISA binding as area under the curve (AUC) of Day 14 serum to SpyCatcher NP and VLP particles, with means and standard deviations represented by rectangles and horizontal lines, respectively. Homotypic strains that were present on the mosaic NPs and heterotypic strains that were not present are indicated by the blue and red rectangles, respectively, above the ELISA data. (PNG) [file pone.0247963.s004.png]

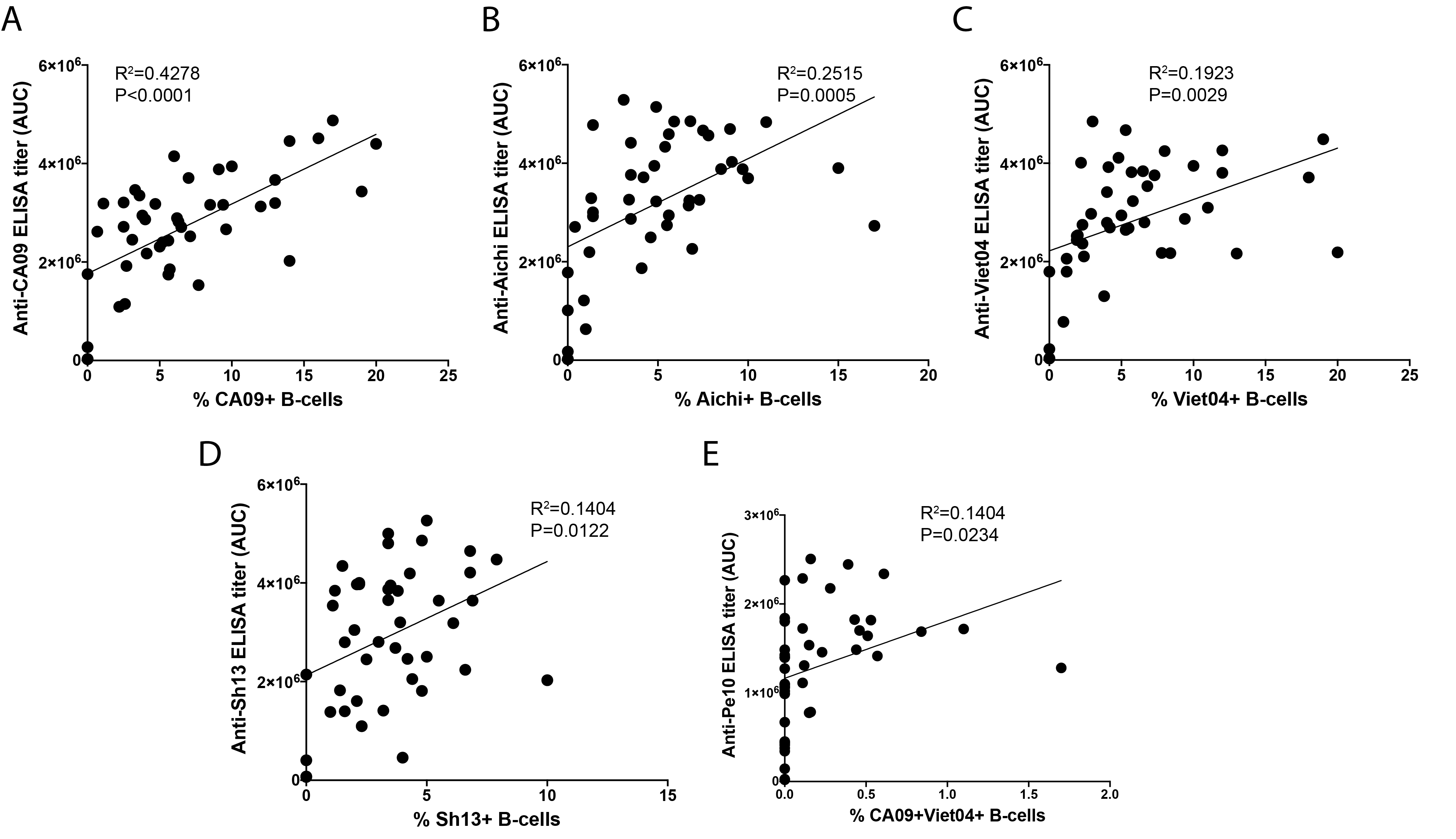

Supplement: S5 Fig — A. Pearson correlation of Day28 CA09+ B-cell population to serum anti-CA09 ELISA AUC titers. B. Pearson correlation of Day28 Aichi+ B-cell population to serum anti-Aichi ELISA AUC titers. C. Pearson correlation of Day28 Viet04+ B-cell population to serum anti-Viet04 ELISA AUC titers. D Pearson correlation of Day28 Sh13+ B-cell population to serum anti-Sh13 ELISA AUC titers. E. Pearson correlation of Day45 CA09+Viet04+ B-cell population to serum anti-Pe10 ELISA AUC titers. (PNG) [file pone.0247963.s005.png]

Figure 1B gel.

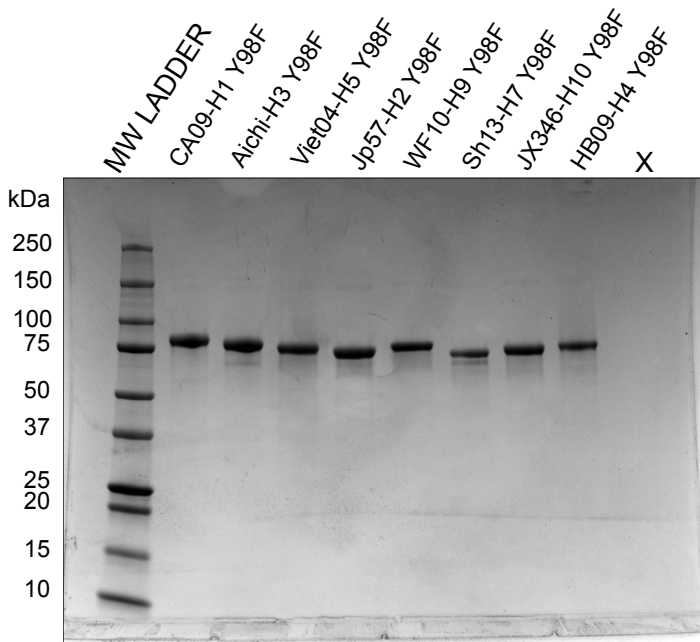

Figure 1D gel left panel.

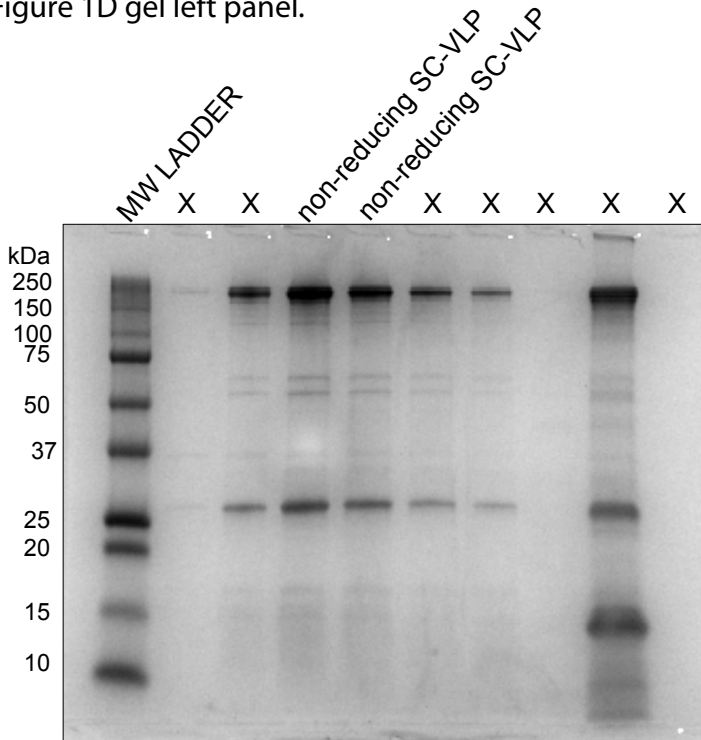

Figure 1D gel right panel.

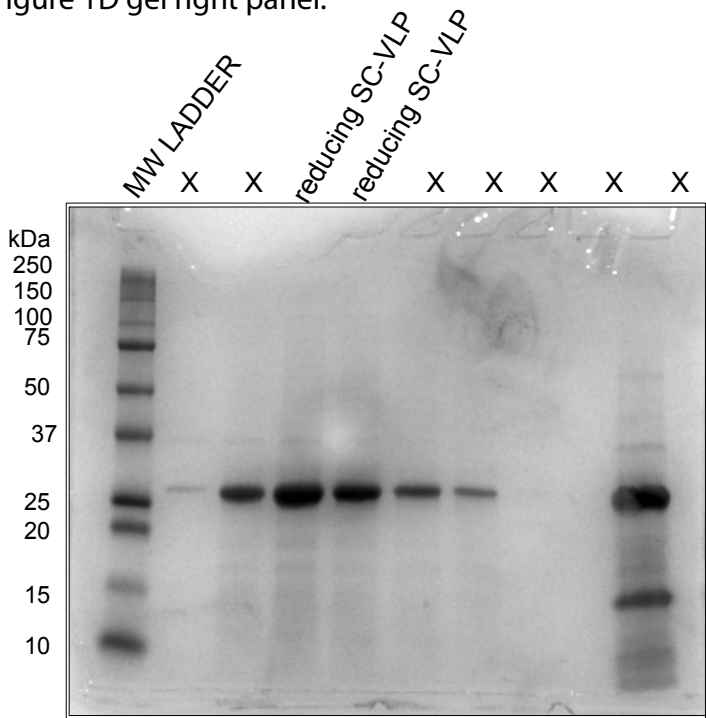

Figure 1F

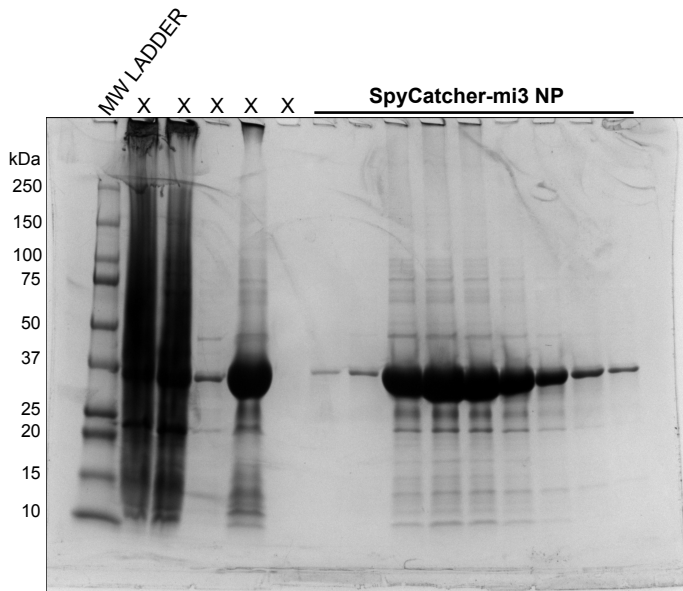

Figure 2B

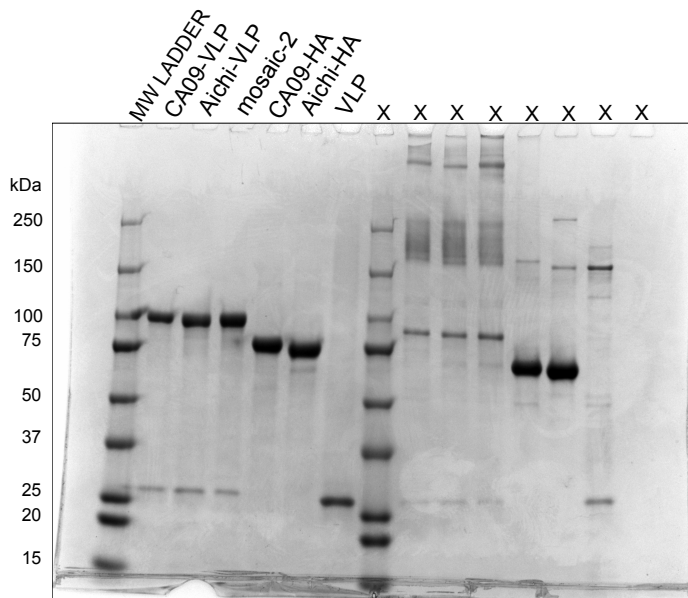

Supplement: S1 Raw images — (ZIP) [file pone.0247963.s007.zip › S1_Raw images_1.pdf]
