# Supplementary material for: Construction, characterization, and immunization of nanoparticles that display a diverse array of influenza HA trimers
Source: PLoS One. 2021 Mar 4;16(3):e0247963. doi: 10.1371/journal.pone.0247963 (PMC7932532; doi:10.1371/journal.pone.0247963)

Figure 2D right gel and Figure 2E gel

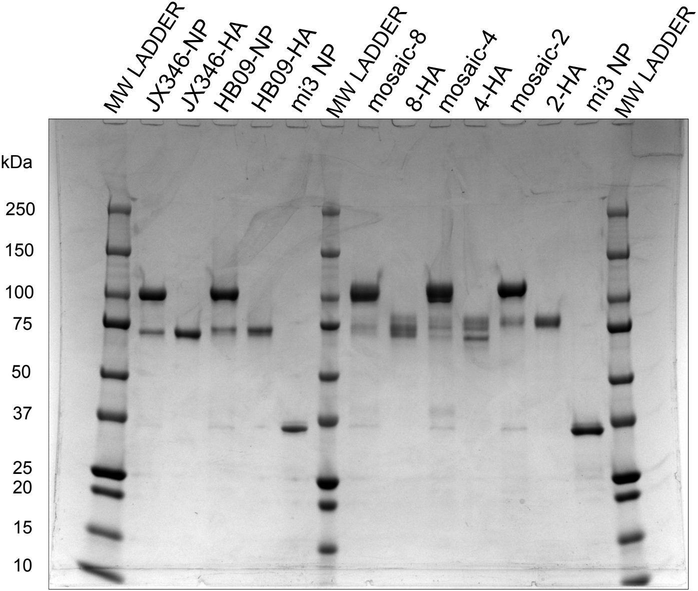

Figure 2D left gel

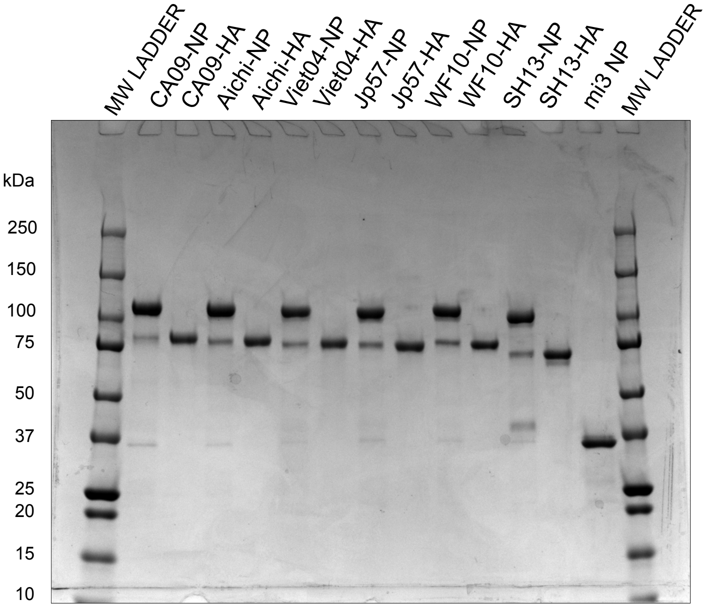

S4B gel bottom panel.

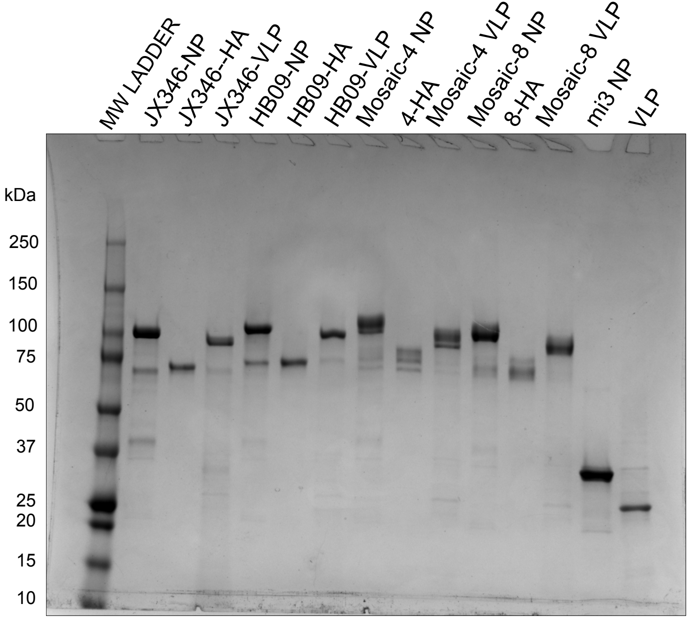

S4B gel middle panel.

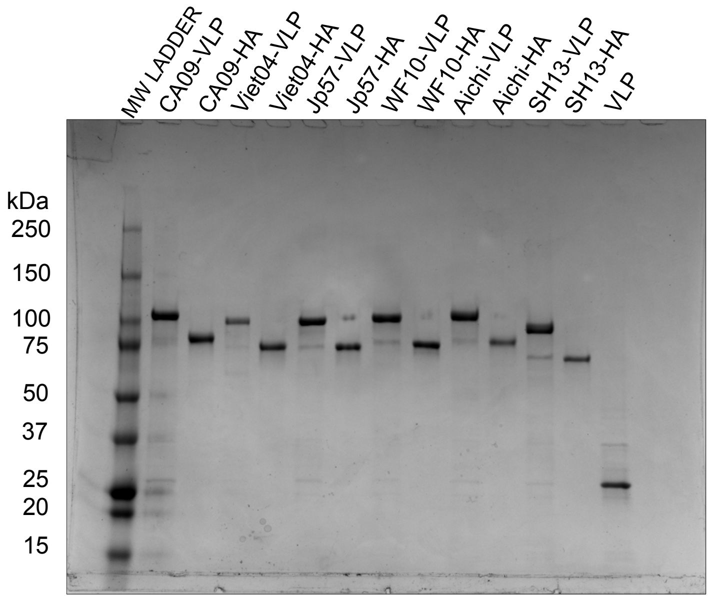

S4B gel top panel.

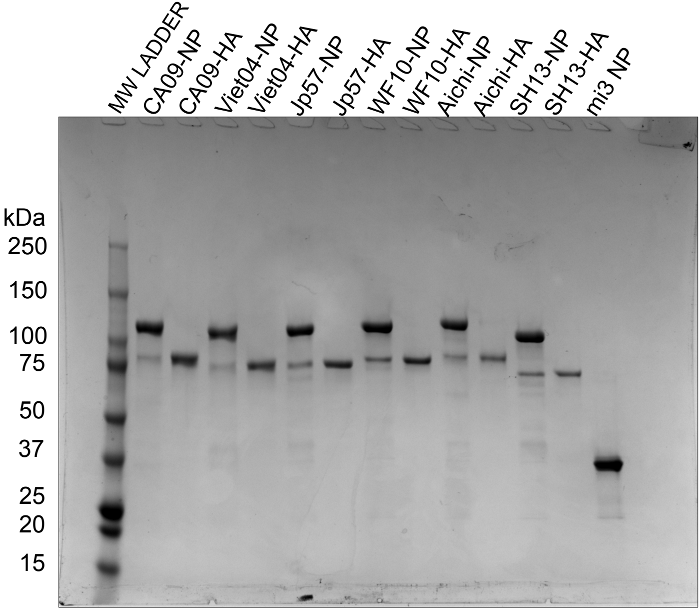

Supplement: S1 Raw images — (ZIP) [file pone.0247963.s007.zip › S1_Raw images_2.pdf]
