# Supplementary material for: Construction, characterization, and immunization of nanoparticles that display a diverse array of influenza HA trimers
Source: PLoS One. 2021 Mar 4;16(3):e0247963. doi: 10.1371/journal.pone.0247963 (PMC7932532; doi:10.1371/journal.pone.0247963)
Supplement: Attachment — Submitted filename: Mosaic-HA Rebuttal v2.docx [file pone.0247963.s010.docx]

January 15, 2021

Dear Victor,

Thank you for sending the reviews of our paper, “**Construction, characterization, and immunization of nanoparticles that display a diverse array of influenza HA trimers**

.” The reviewers have made constructive suggestions, which we have addressed as described below.

*Reviewer #1: This excellent manuscript by Cohen et al. describes nice “Plug and Display” virus like particle (VLP) and nanoparticle (NP) vaccine platforms to present trimeric HA proteins. Both platforms are immunogenic and allow quick generation of mosaic particles displaying HAs of different strains, while the NP platform shows improved yields. The group was able to generate VLPs and NP with 2, 4 and 8 HA valency aiming to target the cross-reactive epitopes on different HA strains. As truthfully reported by the authors, this mosaic strategy did not successfully induce cross-reactive antibodies or B-cells. This, on the other hand, seems to support the hypothesis that an immunodominant hierarchy hinders the induction of cross-reactive antibodies, in which the variable epitopes (typically in the globular head domain) are more accessible than the conserved epitopes in the stem by the BCR, regardless of number of epitopes that are presented at the same time. Nevertheless, this study shows promising vaccine platforms that might be more advantageous when used as an improved seasonal vaccine instead of a universal influenza virus vaccine.*

We thank the reviewer for his/her appreciation of our paper and results.

*1. The HA-VLP in Figure 7 appears to elicit higher antibody responses. The authors hypothesized that this could due to the self-adjuvant effect of the VLPs. However, Page 14, line 2 “The HAs for the SpyCatcher-mi3 conjugations include the sialic acid binding knockout mutation Y98F”; This gives the impression that HAs conjugated with VLP do not have the Y98F mutation. If that is the case, ELISAs performed using recombinant HA without the Y98F could give different results for the two platforms. The authors should provide more clarification of whether the wt or mutant HA were used in the VLP platform and the ELISAs.*

All of the mi3 and VLP conjugations with HA, apart from those in Figure 4, were carried using the HA Y98F mutant. This is because particles conjugated with wt HA precipitated, likely due to HAs with an intact sialic acid binding site interacting with sialylated HAs on neighboring nanoparticles with high avidity, and therby promoting promoted aggregation. We initially tried to use HAs expressed in kifunensine-treated cells, which would result in high mannose N-glycans without sialic acids, but we had variable success with producing HAs that completely lacked sialylated N-glycans. Therefore both the HA-VLP and HA-mi3 in Figure 7 were prepared with the HA Y98F mutant. However, all ELISAs were carried out with wt HA. This is now clarified in the revised Figure 4 and Figure 7 legends and in the Methods.

*2. Is the SpyCatcher immunogenic? Have the author measured antibodies specific to the Spycatcher or the NP/VLP separately?*

As reported in Howarth et al. (2020), SpyCatcher is immunogenic. We verified this finding as shown in the revised manuscript, supplementary figure 4 Figure 5B, which shows ELISA binding of serum from HA-mi3–immunized mice to SpyCatcher-mi3 nanoparticles.

*3. The immunization route and adjuvant used in Fig 4 and 5 are different from those used in Fig 7. Can the author explain a little bit more why that is?*

The immunization route was the same for all of the animal experiments. The adjuvant for Figure 4 and 5 was also the same. We used a different adjuvant for the experiments in Figure 7 in order to compare to experiments shown in Kanekiyo et al., 2019 and Barnum-Boyoglu et al., 2020.

*Reviewer #2: The manuscript by Cohen et al. demonstrates that in two different nanoparticle formulations (AP205 VLPs and mi3) carrying multiple strains of influenza HA, there are no major differences in antibody responses between antigen (HA trimers) presented as mosaic particles or admixed particles. Although the results are "negative" per se, the manuscript provides valuable insights for the field and kudos to the authors for such a through work. However, prior to publications several questions should be addressed and some new data needs to presented.*

We thank the reviewer for his/her appreciation of our paper.

*1. The choice of i.p delivery of the vaccine in mice is interesting but unusual. Why was i.p. chosen as the route? Typically intramuscular, intradermal/subcutaneous, or intranasal routes are chosen.*

The IP immunization route was necessary to deliver the desired volume for each injection.

*2. Please provide statistics in the figures. There are discussions of the stats in the text, but at least each figure or figure legend should summarize the statistics for the corresponding data.*

In the revised figures, we have added statistics for some of the pairwise combinations. To avoid making the figures uninterpretable, we did not show statistics for all pairwise combinations.

*3. Do the authors have any data on the germinal center reaction for these groups? It is critical to understand, from an Ab response point of view, whether the GC responses were all similar or whether these formulations failed to elicit strong GC responses. This would provide mechanistic insights better than just looking at Ab responses.*

We agree with the reviewer that it would be very interesting to examine GC responses. Unfortunately, we do not have appropriate samples from the immunized mice at this time, so doing these experiments would require repeating the entire immunization series in mice, which would be both expensive and time-consuming. We note that the other papers examining responses to mosaic HA nanoparticles that we cited (Kanekiyo et al., 2019 and Boyoglu-Barnum et al., 2020) did not include GC studies, thus suggesting that these experiments are beyond the scope of our paper and the previous studies.

*4. It is disappointing that no T cell response data were shown. It would much to the study if lymph nod eor splenic T cell assays are performed and reported.*

Again, we agree with the reviewer that it would be interesting to examine T cell responses. However, we no longer have lymph node or spleen samples from the immunized mice, and note that T cell assays were not reported in other papers reporting mosaic HA nanoparticles (Kanekiyo et al., 2019 and Boyoglu-Barnum et al., 2020).

We thank the reviewers for their careful reading of our paper and excellent suggestions, which we believe have improved the revised manuscript.

Sincerely, Pamela J. Bjorkman
